# Supplementary material for: Association between oral microbiome and seven types of cancers in East Asian population: a two-sample Mendelian randomization analysis
Source: Front Mol Biosci. 2023 Nov 21;10:1327893. doi: 10.3389/fmolb.2023.1327893 (PMC10702768; doi:10.3389/fmolb.2023.1327893)
Supplement: Supplementary file 1 [file DataSheet1.ZIP › Supplementary Materials/MR plots of saliva/saliva═╝/Gastric cancer/pheno.581_to_gastric cancer_forest.pdf]

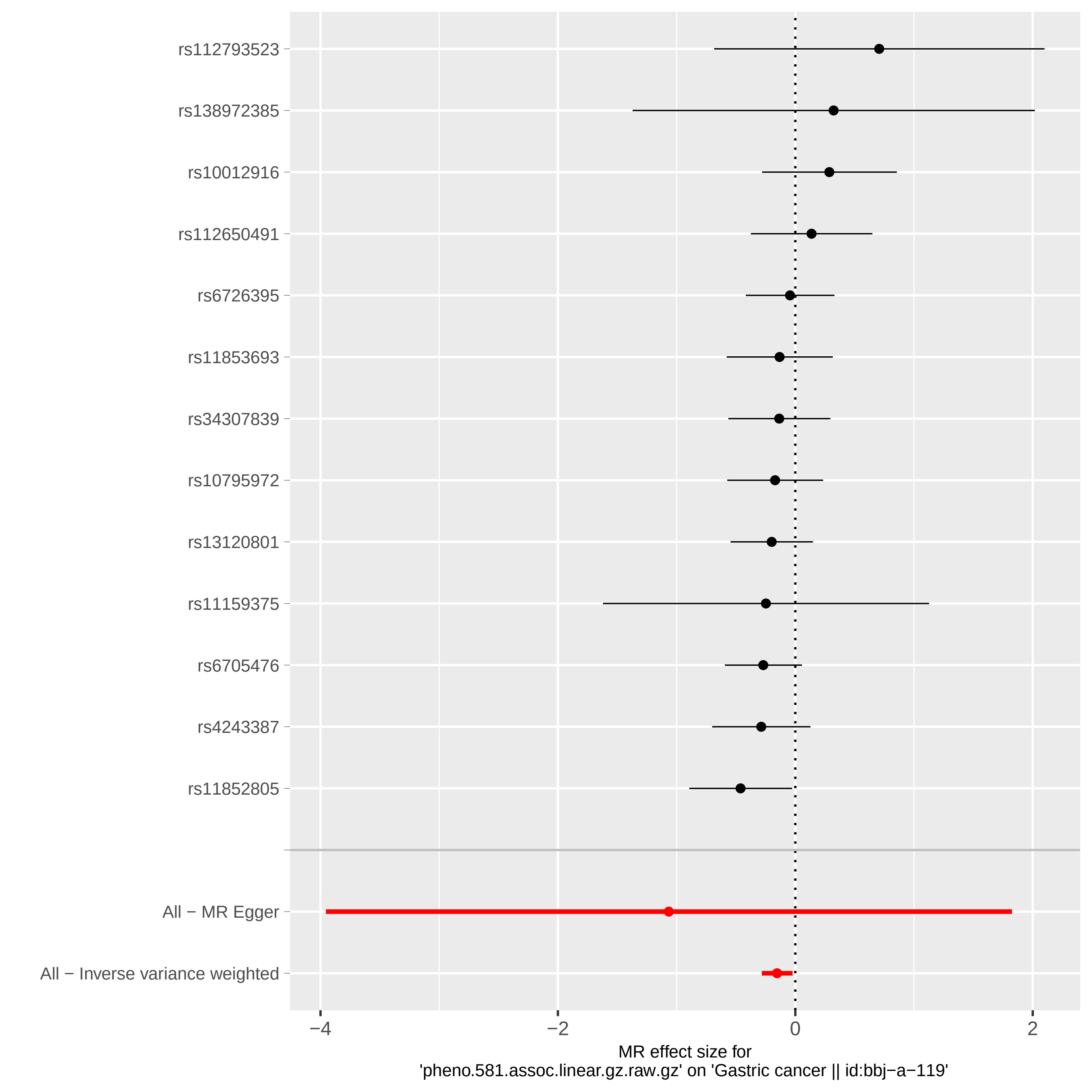
rs112793523
rs138972385
rs10012916
rs112650491
rs6726395
rs11853693
rs34307839
rs10795972
rs13120801
rs11159375
rs6705476
rs4243387
rs11852805
All − MR Egger
All − Inverse variance weighted
−4
−2
0
2
MR effect size for
'pheno.581.assoc.linear.gz.raw.gz' on 'Gastric cancer || id:bbj−a−119'
